# Methane by the Numbers: The Need for Clear and Comparable Methane Intensity Metrics

**DOI:** 10.1021/acs.est.5c13990

**Published:** 2026-03-09

**Authors:** Matthew R. Johnson, Bradley M. Conrad, Daniel J. Zimmerle, Robert L. Kleinberg

**Affiliations:** † Energy & Emissions Research Lab (EERL), 6339 Carleton University, Ottawa, ON Canada, K1S 5B6; ‡ Methane Emissions Technology Evaluation Center, Colorado State University, Fort Collins, Colorado 80524, United States; § Columbia University Center on Global Energy Policy, New York, New York 10027, United States

**Keywords:** Methane intensity, methane metrics, loss rate, embodied emissions, oil and gas, MRV, emissions reporting

## Abstract

Global efforts to track methane emissions from oil and natural gas operations have recently converged around measures of methane emissions intensity, including emergent requirements for reporting as part of import standards. However, multiple definitions of methane intensity have led to conflicting approaches that hinder clear comparisons among regions and obstruct the development of effective policy. This study analyzes six of the predominant methane intensity metrics and shows how half, by attributing methane exclusively to gas production while overlooking coproduced oil and liquids, can bias comparisons among jurisdictions and have limited practical utility. These simple loss rates are strongly dependent on gas-oil ratios and tend toward meaningless infinite methane intensities in oil-dominant operations. The three remaining metrics overcome this limitation and are recommended as unbiased and directly intercomparable measures of methane performance. We further show how these latter metrics, which effectively benchmark methane emissions against total energy production, are computationally and functionally equivalent when emissions are allocated to oil and gas operations using energy production. Finally, we address the challenge of propagating emissions through the supply chain and demonstrate how, for the recommended intensity metrics, embodied intensities of any facility’s outputs can be easily calculated from feeder-facility intensities and energy production.

## Graphical abstract

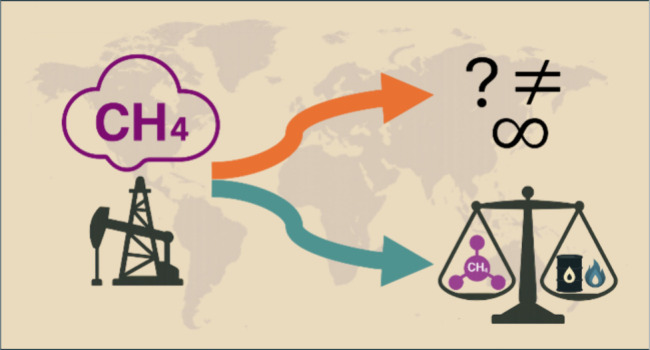

## Introduction

As global efforts to reduce oil and gas sector methane emissions intensify, the need for robust, transparent, and standardized methane intensity metrics has become increasingly apparent. These metrics, which in broad terms quantify methane emissions relative to a unit of energy produced or physical output, are essential for benchmarking performance, informing policy, and driving mitigation. In the best case, an objectively and consistently defined methane intensity metric enables comparisons across companies, regions, and timeframes, offering a pathway to greater transparency and accountability. Clear and comparable metrics are the essential underpinning of proposed import standards within new EU methane regulations,^1^ liquified natural gas (LNG) buyer-led initiatives such as the Coalition for LNG Emission Abatement toward Net-zero (CLEAN),^2,3^ and various independent gas certification efforts.^4–8^

However, the application of methane intensity metrics is fraught with methodological challenges, including multiple definitions, inconsistent terminology, and differing bases.^9^ The aim of this paper is to bring clarity to a range of common methane intensity metrics and how they relate, discuss advantages and limitations, share recommendations for best practice use of methane metrics in measurement, reporting, and verification (MRV), and show how energy-based metrics enable simple and robust calculations of methane intensities throughout oil and gas supply chains.

## Methane Emission Metrics

Common methane emission metrics can be generalized under one of six definitions, as detailed in Table 1. While these metrics each relate methane emissions to production in some way, they differ in whether total methane or methane associated with just gas production is considered; whether total production, only gas production, or only methane within gas production is considered; and whether the ratios are defined on a mass, volumetric, or energy basis. Confusingly, all may colloquially be referred to as “methane intensity”. Additionally, metrics may be calculated using produced oil and gas or marketed oil and gas, although metrics based on marketed outputs are most indicative of emission impacts at the point of consumption. Notably, marketed data are also generally more reliable as they are often measured by both the supplier and the receiver at multiple points along the supply chain. By contrast, particularly at oil and heavy oil sites, unmarketed gas production may only be estimated as further discussed below. Since marketed volumes are also more commonly available across jurisdictions, we subsequently use marketed outputs as the normalizing basis.

**Table 1:** Methane Emission Metrics and Their Equivalencies

Metric	Conceptual Definition	Calculation^a^ and Relation to EF_CH_4__	Advantages/*Disadvantages*
Methane Emission Factor, EF_CH_4__ [g/MJ] (MiQ,^6^ Colorado^b^,^18^ U.S. WEC^19^)	Mass of emitted methane per marketed energy	${\mathrm{EF}}_{CH_{4}}=\frac{m_{CH_{4}}}{E_{\mathrm{oil}}+E_{\mathrm{gas}}}$	• A concise, easily calculated emission to benefit ratio – methane emissions per unit of delivered energy
			• *Lacks units of % that are often favorable in policy; not interpretable as a “loss rate”*
			
Methane Energy Intensity, *MI*_*e*_ [%]	Energy of emitted methane per marketed energy	MIe=mCH4HHVCH4ρCH4(Eoil+Egas)=EFCH4HHVCH4ρCH4	• Interpretable as% of total energy lost as methane
			• Equally interpretable as% of energy lost with emitted methane from gas production^c^
			• Directly related to EF_CH_4__ via constant scaling factor (HHV_CH_4__/ρ_CH_4__)
			
Methane Intensity (true “Loss Rate”), MI_LR_ [%] (NGSI,^13^ MiQ,^5^ OneFuture,^7^ Veritas^8^)	Emitted methane from gas per methane in marketed gas	MILR=mCH4EgasρCH4(Eoil+Egas)XCH4,gasVgas=EFCH4HHVgasρCH4XCH4,gas	• Interpretable as% of gas (or methane) emitted prior to reaching market
			• A methane “loss rate” that can be consistently applied across oil and gas sites
			• *Varies with methane fraction in gas (X*_*CH4,gas*_ *) that is rarely publicly available and may vary among basins and along the production chain which complicates calculation, but otherwise directly relatable to EF*_*CH*_4__
			
*Not recommended/Potentially misleading when comparing among basins or operators*			
Methane to Whole Gas Ratio (simple “Loss Rate”), MGR_wg_ [%] (OGCI,^20^ U.S. WEC^19^)	Total volume of emitted methane per volume of marketed gas	MGRwg=mCH4ρCH4Vgas=EFCH4HHVgas(Eoil+Egas)ρCH4Egas	• Simple to calculate
			• *Not comparable among basins or operators as it varies with relative production of gas and oil*
			• *Not accurately a loss rate but often interpreted as one*
			• *Becomes infinite as gas production goes to zero*
			• *For the same g/MJ methane emissions, penalizes operators who produce oil*
			• *Mixes methane and whole gas*
			
Methane to Gas Energy Ratio, MGR_e_ [%]	Energy in total emitted methane per energy in marketed gas	MGRe=mCH4HHVCH4ρCH4Egas=EFCH4HHVCH4(Eoil+Egas)ρCH4Egas	• *Not comparable among basins or operators as it varies with relative production of gas and oil*
			• *Becomes infinite as gas production goes to zero*
			• *For the same g/MJ methane emissions, penalizes operators who produce oil*
			
Methane to Gas Methane Ratio (simple “Methane Loss Rate”), MGR_CH_4__ [%]	Total emitted methane per methane in marketed gas	MGRCH4=mCH4ρCH4XCH4,gasVgas=EFCH4HHVgas(Eoil+Egas)ρCH4XCH4,gasEgas	• *Not comparable among basins or operators as it varies with relative production of gas and oil*
			• *Becomes infinite as gas production goes to zero*
			• *For the same g/MJ methane emissions, penalizes operators who produce oil*
			• *Varies with methane fraction in gas (X_CH_**4**_,gas_) that is rarely publicly available and varies among basins and along the production chain which complicates calculation*

a Equation variables are defined as follows: *m*_CH_4__ is the mass of emitted methane [g]; *E*_oil_ is the energy content of marketed oil and condensate [MJ]; *E*_gas_ is the energy content of marketed gas [MJ]; ρ_CH_4__ is the density of pure methane at oil and gas industry standard conditions of 15 °C and 101.325 kPa [679.83 g/m^3^]; HHV_CH_4__ is the volumetric higher heating value of pure methane [37.7044 MJ/m^3^]; HHV_gas_ is the volumetric higher heating value of marketed gas [MJ/m^3^]; *X*_CH_4_,gas_ is the mole or volume fraction of methane in marketed gas [*m*_CH_4__^3^/*m*_gas_^3^].

b Colorado Regulation Number 7 specifies greenhouse gas intensity targets in terms of carbon dioxide equivalent (including contributions from carbon dioxide, methane, and nitrous oxide) per unit energy in units of barrels of oil equivalent.

c If total methane emissions are allocated to marketed gas and oil on an energy basis, then ${\mathrm{MI}}_{e,\mathrm{gas}}=\frac{m_{CH_{4}}\left(E_{\mathrm{gas}}/\left(E_{\mathrm{oil}}+E_{\mathrm{gas}}\right)\right){\mathrm{HHV}}_{{\mathrm{CH}}_{4}}}{\rho_{{\mathrm{CH}}_{4}}E_{\mathrm{gas}}}={\mathrm{MI}}_{e}$

### Methane Emission Factor, EF_CH_4__ [g/MJ]

As the primary mission of the oil and gas industry is to produce hydrocarbons that are valued for their energy content, methane emissions per unit of marketed energy—defined here as the methane emission factor, EF_CH_4__ [g/MJ]—is defensibly what ultimately matters. Beyond oil and gas, this simple metric also permits direct comparisons across sectors including, for example, relative methane emissions per delivered energy from hydropower^10,11^ or from coal production.^12^ However, as summarized in Table 1, despite being a concise emission-to-benefit metric, EF_CH_4__ lacks the percentage units that are favorable in policy communications and is not readily interpretable as a “loss rate” during gas and/or oil production.

### Methane Energy Intensity, MI_e_ [%]

A simple alternative is to define the methane energy intensity, MI_e_, which considers the energy content of emitted methane per unit of delivered energy. This gives a unitless finite ratio that, except in extreme cases, is bounded between zero and one, is readily reported in units of %, and represents the fraction of delivered energy lost due to emitted methane. Notably MI_e_ is directly related to EF_CH_4__ via a constant scaling factor (the mass-based higher heating value of methane, HHV_CH_4__/ρ_CH_4__ = 0.055571 MJ/g) such that the two metrics are interchangeable. Moreover, if total methane emissions are allocated between oil and gas based on energy production as further discussed below, then MI_e_ is equally interpretable as the percentage of energy lost as methane from gas production (see Table 1 footnotes), whether from dry gas (predominantly gas) or associated gas (gas produced in concert with oil or condensate) production.

### Methane Intensity, MI_LR_ [%]

The methane intensity or true “Loss Rate”, MI_LR_ [%], as defined by the Natural Gas Sustainability Initiative (NGSI)^13^ and similarly used by MiQ,^5^ OneFuture,^7^ and Veritas,^8^ nominally quantifies the percentage of marketed natural gas that is emitted prior to reaching market. This is conceptually the easiest metric to communicate in public or policy forums, which is a significant advantage. Moreover, as shown in the equations within Table 1, if total methane emissions are allocated to oil and gas based on their relative amounts of marketed energy, then MI_LR_ scales directly with EF_CH_4__ (scaled by the gas heating value, volume fraction of methane in gas, and methane density at standard conditions) and is readily calculable for any site or facility regardless of whether it handles gas and/or oil. This means that MI_LR_ is useful as a single loss rate metric that can be consistently applied across oil and gas sites.

### Energy Allocation of Emissions

As noted by Allen et al. “energy allocation [of methane emissions] is a rational choice given that the products are valued for their energy content”.^14^ As an indication of the prevalence of an energy basis, most trading of natural gas products, such as the Henry Hub spot price, is calculated on an energy basis. Moreover, given that hydrocarbon wells typically produce a mixture of gas, oil, and water, this approach is often the only logical option for many methane sources, which cannot be easily attributed to oil or to gas alone. Most obviously, this includes on-site separators and related equipment that by design handle mixed fluids and can be significant aggregate methane emitters in aerial surveys.^15,16^ Methane emissions from these units are inherently tied to the combined flow of oil and gas. Similarly, at sites handling both oil and gas, methane emissions from flares, heaters, generators, drilling, servicing, and maintenance activities are also byproducts of joint oil and gas production. This reality is reflected in MiQ guidelines which suggest that methane emissions from all sources other than gas dehydrator vents and those specifically related to compressors be “energy-allocated” to oil and gas.^5^ Although, mathematically, energy allocation implies that EF_CH_4__ is equal for oil and gas, available inventories from satellite-based flux inversions that attempted to derive separate oil and gas methane emission factors suggest that this is empirically justified. Specifically, using combined data for 33 countries from Shen et al.^12^ and Chen et al.,^17^ the production-weighted average methane emission factor was 0.17 g/MJ for oil and 0.22 g/MJ for gas, or approximately 0.2 g/MJ in both cases considering the likely uncertainty in these estimates.

An important consequence of attributing methane emissions based on energy is that the three primary metrics, EF_CH_4__, MI_e_, and MI_LR_, are directly related via multiplicative scaling factors. This means they are ultimately interchangeable provided that separate oil and gas production data, heating values, and gas methane fractions used in calculations are reported; we incidentally recommend that this be a default requirement for all intensity reporting. Production data^21,22^ and heating value data^23–25^ are generally readily findable. However, the mole (or volume) fraction of methane in delivered gas (needed for MI_LR_ specifically) is, in the authors’ experience, harder to source. Although methane fraction in gas at upstream sites in particular can vary widely from approximately 0.4–0.98,^26–29^ this is not a critical issue so long as the value used in calculations is reported. Many recent studies^12,30–32^ commonly assume a mole fraction 0.9, which could serve as a default reference. This interchangeability means it is possible to simultaneously plot data in terms of all three metrics within a single figure, as demonstrated in Figure 1b.

**Figure 1:**
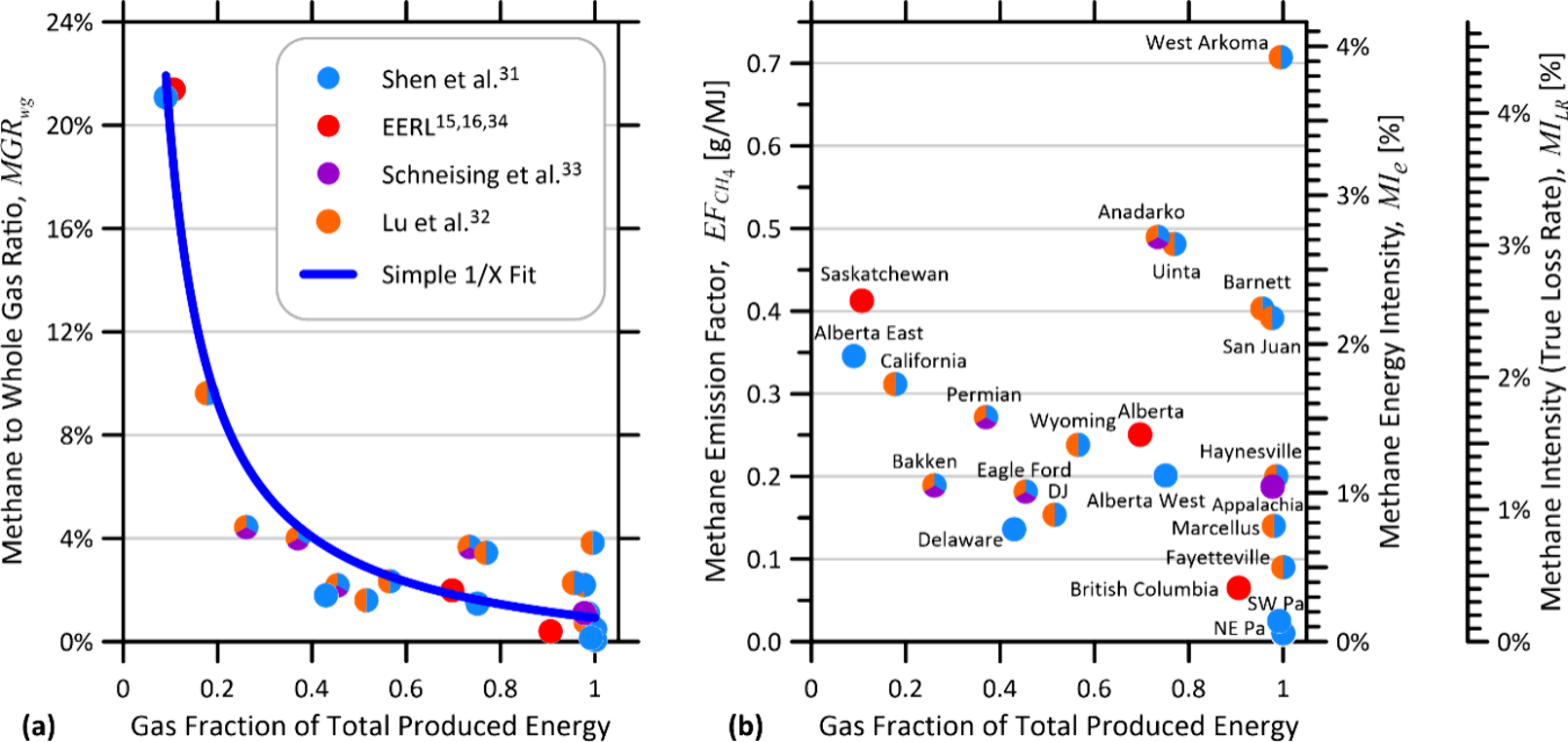
(a) Measured methane to whole gas ratios (MGR_wg_) for North American oil and gas basins derived from published satellite and aerial studies.^15,16,31–34^ Reciprocal fit reveals that MGR_wg_ is primarily dependent on the relative fractions of oil and gas produced within a basin and is not comparable among basins. (b) Methane Emission Factor (EF_CH_4__ [g/MJ]) and Methane Energy Intensity (MI_e_ [%]) show no trends with gas fraction of produced energy and instead reveal relevant methane performance differences among basins. Rightmost axes also show how MI_LR_ scales directly with EF_CH_4__ and MI_e_ when assuming a common gas heating value and methane fraction (HHV_gas_ = 38.169 MJ/m^3^ and *X*_CH_4__ = 0.9 in this case).

### “Loss Rate” Metrics that Attribute All Methane Emissions to Gas Production

The remaining three metrics in Table 1—Methane to Whole Gas Ratio (commonly termed the “Loss Rate” or what the Oil & Gas Climate Institute (OGCI) defines as “methane intensity”^20^), MGR_wg_, Methane to Gas Energy Ratio, MGR_e_, and Methane to Gas Methane Ratio (sometimes termed the “Methane Loss Rate”), MGR_CH_4__—differ in that they implicitly assign all methane emissions to natural gas. Allen et al.^14^ have previously highlighted problems with this approach, particularly in regions where both oil and gas are produced (which includes all major basins in North America). In addition to being inconsistent with Life Cycle Analysis principles, assigning all methane emissions to natural gas production inherently and unrealistically implies that there are zero methane emissions associated with oil production. This can further lead to double counting of emissions should oil be subsequently assigned methane emissions (e.g., as part of a supply chain calculation, as further discussed below) in a region where all emissions have previously been assigned to natural gas.

Inspecting the definitions of MGR_wg_, MGR_e_, and MGR_CH_4__, reveals that these metrics are also unbounded and all tend to infinity as gas production approaches zero. While separating out the mass of gas produced seems intuitive, this separation ultimately means that the resulting metric is not a “loss rate” but an indirect, and imprecise, representation of gas-oil ratio in the region where they are calculated. This is apparent in Figure 1a which plots *MGR*_*wg*_ (simple “loss rate” or “OGCI methane intensity”) for North American oil and gas basins from recent measurements^15,16,31–34^ and reveals how the data follow a simple reciprocal fit of the fraction of marketed energy in the form of natural gas. This problematic dependence means that MGR_wg_, MGR_e_, and MGR_CH_4__ are not directly comparable among different regions with different gas-oil production ratios. Although the scatter about the reciprocal trend identifies meaningful differences in methane performance, these differences are largely obscured by a strong dependence on gas fraction of to total energy, which becomes especially dominant in regions with low gas-oil ratios (i.e., higher relative oil production). It is the authors’ opinion that simple “loss rate” metrics that attribute total emissions solely to gas production are inherently misleading when compared among sites and should be avoided.

At individual sites, MGR metrics can have utility as an environmental indicator representing the fraction of gas lost from the site if defined based on produced rather than marketed gas. However, this definition can also be problematic in practice. First, many upstream sites handle oil and gas without having any gas production at that facility (e.g., treaters, metering facilities, gas plants, etc.). However, these facilities still commonly have reported or measurable emissions. Defining MGR based on produced plus received gas is possible, but this metric ignores the embodied emissions of the received gas unless they are also somehow considered as part of the supply chain as further discussed below. Second, sites using gas lift (artificial lift) or a range of enhanced production methods (e.g., gas cap injection, dispersed gas injection, gas cycling, etc.) reinject natural gas into the reservoir. Particularly at offshore facilities, a majority of extracted gas may be reinjected. At these sites, gas may be ‘produced’ multiple times, rendering gas production an ambiguous and uncertain denominator. Third and most importantly, at many oil production sites, unmarketed gas production is not directly measured. Instead, produced gas is commonly estimated by multiplying infrequently measured gas-oil-ratios (GOR) or gas-in-solution (GIS) values by oil production volumes leading to unreliable estimates, especially at heavy oil sites.^35,36^ Indeed, field studies using a range of measurement approaches have shown multiple examples of oil and heavy oil sites with measurable methane emissions yet zero reported gas production^37,38^ or measured methane emissions that exceed both total reported gas production and total production plus receipts.^36–38^ These factors can lead to misleading comparisons, where, for example, two sites with identical methane emissions (and potentially identical marketed outputs) but different estimates of unmarketed gas production can have significantly different MGR.

By contrast, Figure 1b plots the same methane emissions data^15,16,31–34^ in terms of the first three metrics of Table 1; this plot shows no singular dependence on gas-oil ratio. This implies that these metrics are generally applicable and comparable across different basins, without translation, and most importantly, that the scatter among basins indicates performance variations in the amount of methane emitted per unit of delivered energy. It is worth noting that a recent preprint^9^ comparing EF_CH_4__ relative to MGR_CH_4__ correctly illustrates that simple loss rates favor gas-dominant sites (through scaling with the fraction of marketed energy in the form of natural gas), but incorrectly asserts that EF_CH_4__ favors oil-dominant sites; Table 1 and Figure 1 both show that this is not the case and specifically that EF_CH_4__, MI_e_, and MI_LR_ are all agnostic to gas-oil production ratios.

Finally, it is notable that it is possible to plot the three primary metrics of Table 1—EF_CH_4__, MI_e_, and MI_LR_—using a single set of data points on a single graph. As noted above, EF_CH_4__ and MI_e_ are related by a constant scaling factor. However, if a common reference gas heating value and gas methane fraction are assumed (i.e., HHV_gas_ = 38.169 MJ/m^3^ and *X*_CH_4__ = 0.9 as further discussed below), then these two metrics also scale directly with MI_LR_ as indicated by the rightmost axis.

### Comparison of Recent Methane Intensity Targets on an Equivalent Basis

Using the equations from Table 1, it is possible to compare various published methane targets on a common basis, as illustrated in Figure 2. Targets defined using either MI_LR_ or EF_CH_4__ metrics appear as horizontal lines since they have no inherent dependence on the gas fraction of total produced energy. This includes OneFuture’s 2025 target of 0.28% for gas production (which is a component of an overall production-through-distribution target of 1.00%)^7^ and MiQ’s “Grade A” targets, which specify 0.05% for onshore gas production^5^ and 50 g/BOE for onshore petroleum production^6^ (which converts to 0.00817 g/MJ using their specified oil energy content of 5.8 MMBtu/bbl and is equivalent to a true loss rate of MI_LR_ = 0.0511%, assuming the suggested typical natural gas heating value of 38.169 MJ/m^3^ and methane fraction of 0.9). The horizontal dotted green line represents Colorado’s 2030 greenhouse gas emission target of 6.80 tCO_2_e/kBOE for “Majority Operators”,^18^ which conservatively equates to a loss rate of MI_LR_ = 0.236% assuming only methane is emitted and using the regulation-prescribed methane global warming potential (GWP_100_) of 30 from the IPCC Fifth Assessment Report^39^ and an oil energy content of 5.689 MMBtu/bbl (6.001 GJ/bbl).^40^ In practice, the actual applicable methane target would be lower assuming the operator also emits CO_2_. This ambiguity is one reason separate rather than combined GHG targets are recommended.^41^ Moreover, as noted in the IPCC Sixth Assessment Report, “expressing methane emissions as CO_2_ equivalent emissions using GWP_100_ overstates the effect of constant methane emissions on global surface temperature by a factor of 3–4 ... while understating the effect of any new methane emission source by a factor of 4–5 over the 20 years following the introduction of the new source”.^42,43^

**Figure 2:**
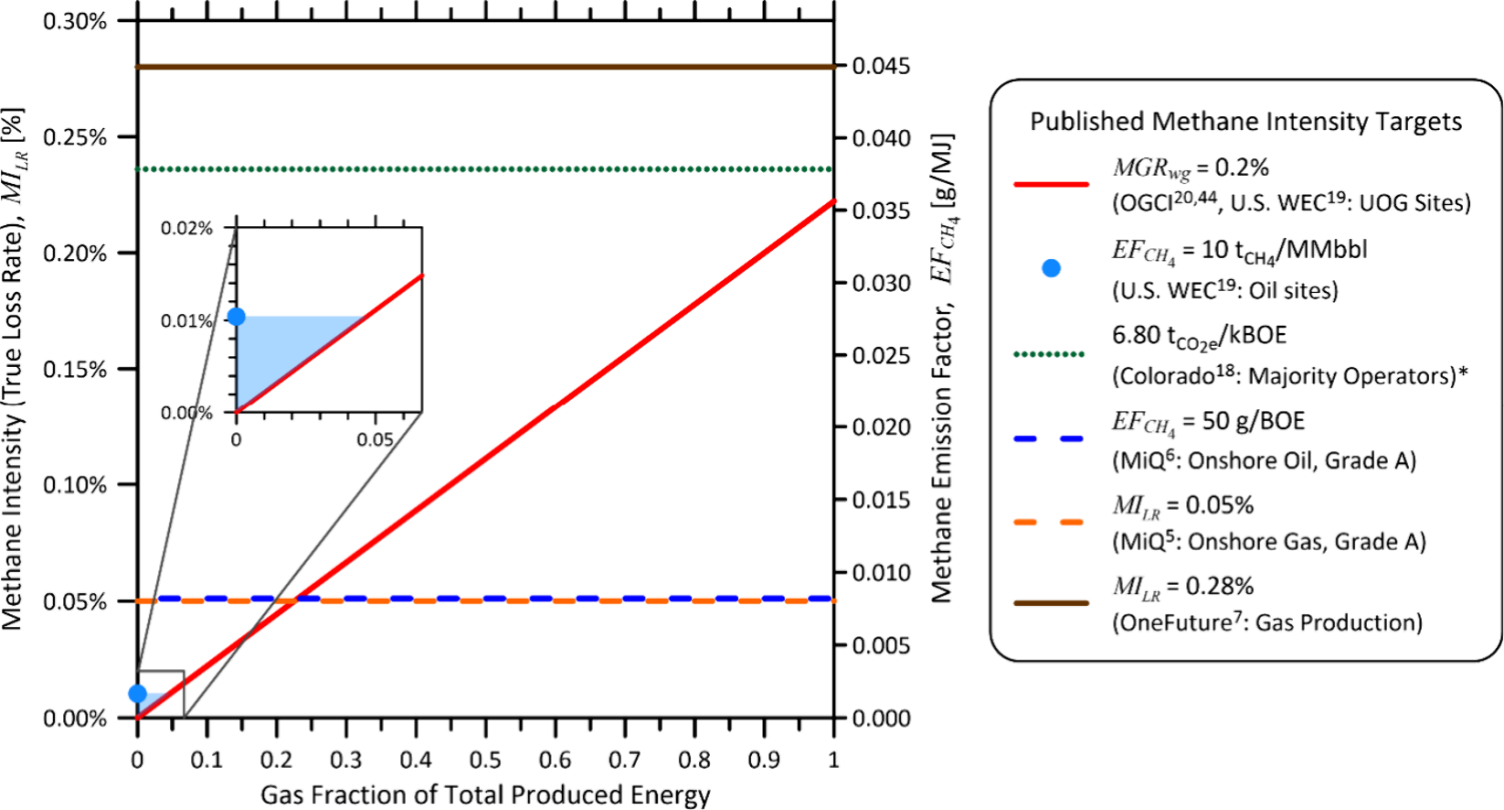
Comparison of various published methane targets converted to a common basis of Methane Intensity (true loss rate), MI_LR_ [%] (left axis) and Methane Emission Factor, EF_CH_4__ [g/MJ] (right axis). Where required, a standard gas heating value of 38.169 MJ/m^3^ and methane fraction of 0.9 is assumed. *Colorado 6.80 t/CO_2_e target for majority operators^18^ is converted assuming only methane is emitted and using the regulation specified methane global warming potential value of 30 from the IPCC Fifth Assessment Report.^39^ Actual methane targets would be lower in practice if the operator also has CO_2_ emissions.

Notably, targets based on *MGR*_*wg*_, which attribute total oil and gas methane emissions to gas alone as discussed above, vary depending on the gas fraction of total energy (inclined red line). This includes the OGCI 2025 “intensity” target of 0.2% ^20,44^ and the threshold for the onset of methane fees at petroleum and natural gas facilities under the U.S. Waste Energy Charge (WEC),^19^ which is currently slated to commence in 2035.^45^ As the gas fraction of total energy goes to zero such that MGR_wg_ tends to infinity, a target defined with MGR_wg_ necessarily implies that no methane emissions are permitted at sites without gas production, even though the oil stabilization process often emits methane. This issue was presumably recognized during the development of the WEC rules, which specify a separate limit of 10 t_CH4_/MMbbl (equivalent to MI_LR_ of 0.01% or EF_CH_4__ of 0.0017 g/MJ) for sites that send “no natural gas to sale”.^19^ Curiously, these two different limits create a theoretical disincentive to mitigation at sites where gas represents less than 4.7% of total produced energy from oil and gas (see the blue region in the inset axes of Figure 2). In principle, sites in this range could be paradoxically incentivized to simply cease gas conservation to access a higher methane limit without incurrence of fees. While this is unlikely to be a significant issue in practice, it highlights the inherent challenge of using *MGR*_*wg*_ as a metric for upstream oil and gas sites.

### Important Considerations when Evaluating Methane Intensities along Supply Chains

Calculating methane intensities across supply chains, where emissions may occur at distinct and often independently operated stages—from upstream extraction and processing to midstream transportation and downstream distribution—can present special challenges and may require site- or operator-specific considerations to ensure accuracy (e.g., ref 46). While segment-specific methane intensities can be estimated, they are not necessarily additive due to differences in system boundaries and allocation practices. Most notably, volumes of oil and gas entering and leaving each segment and potentially the energy content of products may not be constant due to on-site consumption or chemical processing. For example, a midstream gas plant may consume a portion of received gas for operations while also separating out natural gas plant liquids (NGPL, i.e., ethane, propane, butane, pentane, etc.) as a separate product stream and removing impurities (e.g., water, carbon dioxide, and other inert gases). It is possible or even likely that an upstream operator may not know the exact link between their products and those at the end of the supply chain nor specifically how much of their product is required to produce one unit of final marketed product. However, assuming energy allocation of methane emissions as discussed above, then it is still straightforward to calculate the methane emission factor (and hence MI_e_ and MI_LR_) at each stage of the supply chain.

For any facility *j*, receiving energy products from one or more upstream facilities *i*, the total supply chain methane emission factor for its output, EF_CH___4_,*j*_, is calculated as

1 $${\mathrm{EF}}_{CH_{4},j}=\frac{\underset{i}{\sum }\left({\mathrm{EF}}_{CH_{4},i}E_{i}\right)+m_{CH_{4},j}}{E_{j}}$$

where *E*_*i*_ are the energy products received from each upstream facility *i*, EF_CH_4_,*i*_ is the methane emission factor for each facility *i*, *m*_CH_4_,*j*_ is the methane emitted directly by facility *j*, and *E*_*j*_ are the marketed energy products leaving facility *j*. Importantly, equation 1 holds whether facility *j* processes or consumes any portion of the received energy products *E*_*i*_ and/or produces its own separate products that collectively aggregate to its outputs *E*_*j*_. More generally, so long as each facility along the supply chain similarly calculates their methane emission factor using eq 1, then EF_CH_4_,*i*_ represents the cumulative upstream supply chain methane emission factor of each input *E*_*i*_ into facility *j*. This means that eq 1 accurately tracks the methane emissions throughout the supply chain and, specifically, that EF_CH_4_,*j*_ calculated at any point represents the total (cumulative) supply chain emissions up to that point. Finally, even if the mole fraction of methane in gas varies along the supply chain, the methane intensity (MI_LR_) can still be readily calculated at each stage (including at the point of the final consumer) simply by substituting eq 1 into the MI_LR_ equations of Table 1, while using the relevant mole fraction (*X*_CH_4_,gas,_ *_j_*) for that point in the supply chain.

Although energy allocation of emissions is recommended as discussed above, if an alternative allocation approach is desired (e.g., MiQ’s current suggested approach of allocating all emission by energy except for compressor-related and dehydrator vent emissions), then eq 1 can still hold if it is generalized to incorporate separate emission factors for each product, *k* (e.g., gas, oil, NGPL, etc.):

2 $${\mathrm{EF}}_{CH_{4},j,k}=\frac{\underset{i}{\sum }A_{j,i,k}\underset{k}{\sum }\left({\mathrm{EF}}_{CH_{4},i,k}E_{i,k}\right)+m_{CH_{4},j,k}}{E_{j,k}}$$

Here, *m*_CH_4_ *,j,k*_ is the methane emitted directly by facility *j* that is allocated to product *k*, *A*_*j,i,k*_ is facility *j*’s allocation of methane emissions for each product *k* received from facility *i*, *E*_*j,k*_ is the marketed energy leaving facility *j* as product *k*, and *EF*_CH_4_,*j,k*_ is facility *j*’s methane emission factor for product *k*. Finally, for basin-, state-, or national-scale surveys, where the full supply chain is captured within the survey, then eq 1 or (2) is not necessary, and EF_CH_4__, MI_e_, and MI_LR_ can be directly calculated as in Table 1 using all final marketed energy outputs from the basin (specifically including NGPL).

Finally, we note that the numerator of the above equations always translates directly into the mass of methane emissions cumulative over the supply chain up to and including the facility of interest. Therefore, simple modifications of the denominator of these equations can calculate the embodied methane emissions in an end-use product. For example, for a downstream fertilizer plant, *j*, eq 1 provides an accurate representation of methane emissions per mass of fertilizer produced by replacing *E*_*j*_ with the mass of fertilizer produced and noting that *m*_CH_4_,*j*_ is the methane emitted from the fertilizer plant.

## Recommendations

Based on the preceding analysis, metrics that attribute total methane emissions to gas alone should be avoided since they primarily measure the relative proportion of oil and gas production in a region of interest and are not comparable among basins. This includes MGR_wg_ (also known as the OGCI “methane intensity” or gas loss rate), MGR_e_, and MGR_CH_4__ (also known as the methane loss rate). These metrics are especially problematic for supply chain calculations (refer to eq 2), where the simplistic allocation of all emissions to a single product (gas) could lead to artificially low estimates of intensities of other products in the supply chain and/or give rise to a double counting of emissions.

By contrast, methane emission metrics that compare total methane emissions to total oil and gas energy—MGR_CH_4__ [g/MJ] and MI_e_ [%]—or compare attributed methane emissions from gas to marketed gas production—MI_LR_ [%]—are easily calculated, are broadly applicable with no inherent dependence on relative gas and oil production, and are ultimately interchangeable.

For public and policy communications, the Methane Intensity or true “Loss Rate”, MI_LR_ [%], as adopted by NGSI,^13^ MiQ,^5^ OneFuture,^7^ and Veritas,^8^ is advantageous because it conceptually describes the percentage of natural gas or methane that is emitted prior to reaching market. However, because MI_LR_ scales directly with MGR_CH_4__ as demonstrated, it is perhaps even more useful as a single, easy to communicate “loss rate” metric that can be consistently applied across oil and gas sites.

Although country-, state-, and/or basin-level natural gas heating value data (HHV_gas_) to calculate MI_LR_ are generally available^23–25^ similar to oil and gas production data,^21,22^ to the authors’ knowledge the fraction of methane in natural gas by volume (*X*_CH_4__) is not. In the absence of specific data, a commonly used value of *X*_CH_4__ = 0.9 is suggested as a default, along with HHV_gas_ = 38.169 MJ/m^3^ which is the simple average of available country-level data at industry standard conditions of 15 °C and 101.325 kPa.^25^ These values are used in the reference calculations for Figures 1 and 2. To simplify implementation and promote standardization of calculations, Table S1 and S2 of the Supporting Information provides a referenced list of suggested reference values and conversions.

Ultimately, whether MGR_CH_4__, MI_e_, or MI_LR_ (first 3 rows of Table 1) are reported, we strongly recommend that researchers, companies, and analysts commit to also transparently reporting the production volumes, heating values, methane fractions (if applicable), and methane emission totals used in calculations. This will ensure that methane intensity results are always transparently presented and comparable; will enable trivial recalculations using different data or assumptions if desired; and, most importantly, will support easy and accurate calculation of supply chain emissions using eq 1 or eq 2.

## Supplementary Material



## Data Availability

All data in this manuscript are available directly from the cited sources.

## Notes

Tabulated heating value data for use in reference calculations and brief discussion of the importance of accounting for natural gas plant liquids (NGPL) (PDF)
