# Supplementary material for: Methane by the Numbers: The Need for Clear and Comparable Methane Intensity Metrics
Source: Environ Sci Technol. 2026 Mar 9;60(11):8258–65. doi: 10.1021/acs.est.5c13990 (PMC13019660; doi:10.1021/acs.est.5c13990)
Supplement: Supplementary file 1 [file es5c13990_si_001.pdf]

Supporting Information

Methane by the Numbers: The Need for Clear and Comparable Methane Intensity Metrics

Matthew R. Johnson[1,*], Bradley M. Conrad[1], Daniel J. Zimmerle[2], Robert L. Kleinberg[3]

[1]Energy & Emissions Research Lab (EERL), Carleton University, Ottawa, ON, Canada
[2]Methane Emissions Technology Evaluation Center, Colorado State University, Fort Collins, CO, USA
[3]Columbia University Center on Global Energy Policy, New York, NY, USA

[*]To whom correspondence and material requests should be addressed: Matthew.Johnson@carleton.ca; +1-613-520-2600 ext.4039.

File contains 4 pages, 3 tables

Table of Contents

S1 Suggested default values for reference calculations S2

S2 Importance of Natural Gas Plant Liquids (NGPL) S3

S3 References S4

S1 Suggested default values for reference calculations

Table S1: Suggested default higher (gross) heating values (calorific values) of gaseous species at standard conditions of 15°C and 101.325 kPa. For use when site-specific data are not used or not available.

Product	Molar Mass [kg/kmol]	Density [kg/m^3]	Higher (Gross) Heating Value [kJ/kg]	[MJ/m^3]	[BTU/scf]*	Source / Reference
Natural Gas				38.169	1024.43	Calculated as the simple average of produced gas from IEA country-level data during 2019−2023 (IEA, 2025)
U.S. Wet Gas				43.071	1156	U.S. EIA 2023 data (Table B2), (U.S. EIA, 2025c)
U.S. Dry gas				38.600	1036	
Methane	16.0428	0.6798	55571.0	37.7044	1011.94	NIST Chemistry WebBook (REFPROP) (Lemmon et al., 2018)
Ethane	30.069	1.28253	51951.9	66.067	1773.18	
Propane	44.0956	1.89923	50370.1	93.936	2521.17	
n-Butane	58.1222	2.54425	49546.8	121.793	3268.83	
i-Butane	58.1222	2.53328	49388.8	121.404	3258.39	
n-Pentane	72.1488		49046.0			
i-Pentane	72.1488		48950.0			
n-Hexane	86.1754		48717.5			
i-Hexane	86.1754		48629.0			
Heptane	100.202		48474.0			
Hydrogen	2.01588	0.08521	141948.	12.102	324.81	
Ethylene	28.054	1.1941	50336.3	59.722	1602.89	
Propylene	42.08	1.8087	48941.	87.099	2337.67	
Butene	56.106	2.4502	48456.4	114.981	3086.00	

* The International Table BTU is used consistent with (U.S. EIA, 2025a) for which 1 BTU = 1,055.055 852 62 J

Table S2: Suggested default higher (gross) heating values (calorific values) of liquid species if site-specific data are not used or not available.

Product	Higher (Gross) Heating Value (HHV) [MMBtu/bbl]	[GJ/bbl]	[GJ/m^3]†	Source / Reference
Crude Oil	5.688	6.001	37.746	Average of EIA Monthly Energy Review data for 2019−2023 (Table A2) (U.S. EIA, 2025a)
Crude Oil (given API Gravity or Specific Gravity)	Specific gravity (SG) = 141.5 / (131.5 + API gravity)<br>HHV [MMBtu/bbl] = SG * (7.801796 - 1.3213 * SG^2)<br>HHV [GJ/bbl] = HHV [MMBtu/bbl] * 1.05505585262<br>HHV [GJ/m^3] = HHV [GJ/bbl] / 0.1589873			(U.S. EIA, 2025a)
Natural Gas Plant Liquids (NGPL)	3.587	3.784	23.804	Average of EIA Monthly Energy Review data for 2019−2023 (Table A2) (U.S. EIA, 2025a)
Natural Gasoline (29% isopentane, 29% neopentane, 20% normal pentane, 13% normal hexane, 4% cyclohexane, 3% benzene, and 2% toluene)	4.638	4.893	30.778	(U.S. EIA, 2025a)
Kerosene	5.670	5.982	37.627	
Lubricants	6.065	6.399	40.248	
Residual Fuel Oil	6.287	6.633	41.721	

† 1 barrel of oil (bbl) is equal to 0.1589873 m^3 (U.S. EIA, 2025a)

S2 Importance of Natural Gas Plant Liquids (NGPL)

In some cases, a significant part of the value of marketed hydrocarbons are the natural gas plant liquids (NGPL): ethane (C_2H_6), propane (C_3H_8), normal butane and isobutane (C_4H_{10}), and natural gasoline (C_5H_{12} and larger)(U.S. EIA, 2025b). These are separated from pipeline grade natural gas and non-hydrocarbon gases in gas processing plants. In the United States production of NGPL has been growing rapidly since 2010; in 2024 they amounted to 9 percent by volume of marketed gas production (U.S. EIA, 2025a). In wet gas plays such as Bakken, Eagle Ford, and Utica the fraction is larger. For calculations at the basin level, it is important that natural gas plant liquids be included in the marketed energy denominators in the first three entries of Table 1 of the main text.

For calculations at upstream sites, the heating value of the produced wet gas (i.e., the complete gas stream including any higher hydrocarbons that are subsequently removed at a downstream natural gas plant, i.e., NGPL) should be used. Bakken production provides an extreme but not unusual example for the importance of accounting for NGPL. Using the corrected mean raw gas (wet gas)_ composition at wells in the Bakken from Table S3 (raw gas before processing) (Brandt et al., 2016), the corresponding heating value (calculated using NIST REFPROP, Lemmon et al., 2018) is 60.8121 MJ/m^3 (1632.15 BTU/scf). This is 57% higher than the typical U.S. dry gas heating value of 38.6 MJ/m^3 given in Table S1.

Table S3: Mean reported raw gas composition at wells (gas composition before processing) in the Bakken from Table S4 of (Brandt et al., 2016).

Species	Mole Fractions as in (Brandt et al., 2016)	Mole Fractions Normalized to Sum to 1
Methane (C1)	0.4924	0.49090
Ethane (C2)	0.2103	0.20966
Propane (C3)	0.1509	0.15044
n-Butane (n-C4)	0.0506	0.05045
i-Butane (i-C4)	0.0168	0.01675
n-Pentane (n-C5)	0.0126	0.01256
i-Pentane (i-C5)	0.0090	0.00897
Hexane (C6)	0.0165	0.01645
Hydrogen Sulfide (H_2S)	0.00005	0.00005
Carbon Dioxide (CO_2)	0.0070	0.00698
Nitrogen (N_2)	0.0367	0.03659
Argon (Ar)	0.0002	0.00020

Using the mean API Gravity for Bakken oil of 41.93 (Brandt et al., 2016) in conjunction with the formula in Table S2, Bakken crude oil (which includes lease condensate) has an estimated higher heating value (HHV) of 5.648 MMBtu/bbl. The production-weighted mean wet gas-oil ratio is 1273 scf/bbl (Brandt et al., 2016), which means 2.078 MMBtu of wet gas are produced for each barrel of oil. Thus, the total mean produced energy (oil + wet gas, equal to oil + dry gas + NGPL) at Bakken wells is 7.726 MMBtu/bbl. If a typical dry gas heating value of 1036 Btu/scf from Table S1 were instead used in calculations (effectively neglecting produced NGPL), then the total produced energy would be incorrectly underestimated as 6.967 (10% less), leading to a 10% overestimation of EF_{CH_4}, while the ratio of energy in gas (when incorrectly assumed dry) to total energy (neglecting NGPL) would be 30% less, leading to a 30% underestimation of MI_{LR}.

S3 References

Brandt, A.R., Yeskoo, T., McNally, M.S., Vafi, K., Yeh, S., Cai, H., and Wang, M.Q., (2016). Energy intensity and greenhouse gas emissions from tight oil production in the Bakken formation. *Energy & Fuels*. 30:9613–9621.

IEA, (2025). Natural Gas Information - Database Documentation. International Energy Agency (IEA). Available at: https://www.iea.org/data-and-statistics/data-product/oil-information

Lemmon, E.W., Bell, I.H., Huber, M.L., and McLinden, M.O., (2018). NIST Standard Reference Database 23: Reference Fluid Thermodynamic and Transport Properties-REFPROP, Version 10.0.

U.S. EIA, (2025a). Monthly Energy Review. DOE/EIA-0035 (2025/4), United States Energy Information Administration (U.S. EIA). Available at: www.eia.gov/mer

U.S. EIA, (2025b). Hydrocarbon gas liquids explained: Where do hydrocarbon gas liquids come from? https://www.eia.gov/energyexplained/hydrocarbon-gas-liquids/where-do-hydrocarbon-gas-liquids-come-from.php (accessed August 3, 2025)

U.S. EIA, (2025c). Natural Gas Annual. https://www.eia.gov/naturalgas/annual/ (accessed August 3, 2025)
